# Disrupting of IGF2BP3-stabilized HK2 mRNA by MYO16-AS1 competitively binding impairs LUAD migration and invasion

**DOI:** 10.1007/s11010-023-04887-w

**Published:** 2023-12-02

**Authors:** Peiwei Li, Haibo Ge, Jiangfeng Zhao, Yongjia Zhou, Jie Zhou, Peichao Li, Junwen Luo, Wenhao Zhang, Zhongxian Tian, Xiaogang Zhao

**Affiliations:** 1 Institute of Medical Sciences, The Second Hospital of Shandong University, Jinan, China; 2 Department of Thoracic Surgery, The Second Hospital of Shandong University, Shandong University, No. 247 Beiyuan Street, Jinan, 250033 Shandong China; 3 Shandong Engineering Laboratory for Precise Diagnosis and Treatment of Chest Cancer, Key Laboratory of Thoracic Cancer in Universities of Shandong, Jinan, China

**Keywords:** MYO16-AS1, HK2, IGF2BP3, LUAD, Cancer invasion

## Abstract

Since invasive cancer is associated with poor clinical outcomes, exploring the molecular mechanism underlying LUAD progression is crucial to improve the prognosis of patients with advanced disease. Herein, we found that MYO16-AS1 is expressed mainly in lung tissue but is notably downregulated in LUAD tissues. Overexpression of MYO16-AS1 inhibited the migration and invasion of LUAD cells. Mechanistic studies indicated that H3^K27Ac^ modification mediated MYO16-AS1 transcription. Furthermore, we found that MYO16-AS1 competitively bound to the IGF2BP3 protein and in turn reduced IGF2BP3 protein binding to *HK2* mRNA, decreasing *HK2* mRNA stability and inhibiting glucose metabolism reprogramming and LUAD cell invasion in vitro and in vivo. The finding that the MYO16-AS1/IGF2BP3-mediated glucose metabolism reprogramming mechanism regulates HK2 expression provides novel insight into the process of LUAD invasion and suggests that MYO16-AS1 may be a therapeutic target for LUAD.

**Supplementary Information:**

The online version contains supplementary material available at 10.1007/s11010-023-04887-w.

## Introduction

Lung cancer is the leading cause of cancer-related death in the world [1]. Only approximately 20% of lung cancer patients, including patients with both non–small cell lung cancer (NSCLC) and small cell lung cancer, are alive over 5 years after the first diagnosis [2]. Several studies have reported on the recent epidemiology of lung adenocarcinoma in China: the incidence of lung adenocarcinoma in China maintains a trend of continuous increase over the past two decades [3]. Other studies have reported similar findings, with some suggesting that the increase may be even higher in certain regions of the country and certain populations, such as rural areas and consumers of tobacco, respectively. In addition, the diagnosis and treatment of lung cancer have been hampered since the COVID pandemic in 2020, so lung cancer may now have a worse 5-year survival rate [4, 5]. Thus, lung adenocarcinoma remains a major public health challenge in China, with very high morbidity and mortality rates. Ongoing research is focused on identifying new biomarkers and developing targeted therapies to improve the outcomes of patients with lung adenocarcinoma (LUAD).

Long noncoding RNAs (LncRNAs) are noncoding RNAs that are over 200 nucleotides in length [6], and they are well-known for playing vital roles in biological processes in cancer cells, such as cell cycle progression, differentiation, migration, invasion, metastasis, and metabolism [7]. A recent study reported that the lncRNAs-IGFBP4-1 and lncRNA-AC020978 promote lung cancer progression and glycolytic metabolism in cancer cells [8]. Accumulated evidence also shows that lncRNAs are associated with glucose metabolism reprogramming in cancer; for example, lncRNA LINC00160 mediates colorectal cancer and prostate cancer progression [9]. However, these papers reported only the phenotype and the detailed molecular mechanism by which lncRNAs are involved in cancer invasion or glucose metabolism reprogramming in LUAD remains largely unclear. In this project, MYO16-AS1 is a novel lncRNA expressed mainly in lung tissue but dysregulated in LUAD patients. Thus, the biological effect of MYO16-AS1 and related regulatory mechanisms in LUAD urgently need to be addressed.

Hexokinase 2 (HK2) upregulation is involved in the progression of LUAD [9], and it has been identified as an important metabolic function-associated marker for identifying circulating tumor cells in LUAD patient samples [10]. HK2 is a critical enzyme that mediates the first committed step in glucose metabolism by catalyzing the phosphorylation of glucose to yield glucose-6-phosphate [10]. Thus, small molecule inhibitors targeting HK2 were explored as potential targets for cancer therapy [11]. In this study, we investigated the antitumor role of the lncRNA MYO16-AS1 in LUAD. Our results demonstrated that MYO16-AS1 expression is significantly lower in LUAD tissue than in adjacent normal tissue and cancer cell lines than in normal cells and that low expression of MYO16-AS1 is associated with poor prognosis in LUAD patients. Furthermore, we found that the MYO16-AS1/HK2 axis significantly inhibits migration and invasion in vitro and in vivo and attenuates resistance to cisplatin in vivo. These findings suggest that MYO16-AS1 contributes to the inhibition of LUAD progression and may be a promising therapeutic target in LUAD.

## Materials and methods

### Clinical samples

A total of seven pairs of tumor tissues and adjacent normal tissues were collected from patients with LUAD who did not receive chemotherapy or radiotherapy before surgery at The Second Hospital of Shandong University between July 2019 and August 2020, and this study was approved by the hospital's ethics committee (KYLL-2020(KJ)p-0099). The tissues were immediately frozen in liquid nitrogen after surgical resection and independently verified by two pathologists. The postoperative stage was determined based on the 8th edition of the International Union Against Cancer (UICC) tumor -node-metastasis (TNM) classification criteria.

### RNA sequencing and online bioinformatics analysis

Total RNA was extracted using a TRIzol reagent kit (Invitrogen, Carlsbad, CA, USA) according to the manufacturer’s protocol and used for the construction of a cDNA library after ribosomal RNA depletion. Paired-end sequencing of PCR amplicons was conducted using the Illumina NextSeq 500 (2 × 150) platform at Novogene, China. The raw fastq reads were filtered, trimmed, and then aligned to the human reference genome (hg38/GRCh38) and subsequently counted by feature counts depending on “gene id”. All differentially expressed lncRNAs from sequencing results are shown in Supplemental Table 1. Differential expression analysis was performed using the “DESeq2” R package (4.0.2). An adjusted *p*-value of 0.05 and |log2FC|> 1 were set as the thresholds for significant differential expression. The published data for bioinformatics analysis were downloaded from the GEO database (GSE150976). All data were analysed by the R package “limma” or “DEseq2” in R 4.0.2. An adjusted *p*-value of 0.05 and |log2FC|> 1 were set as the thresholds for significant differential expression. Gene set enrichment analysis (GSEA) was dependent on the online bioinformatics website (https://www.gsea-msigdb.org/gsea/index.jsp). Histone modification regions were classified according to the UCSC website.

### Cell culture

BEAS-2B and 16-HBE cell lines were purchased from American Type Culture Collection (ATCC). The lung cancer cell lines H1299, H1975, and H1650, A549 were obtained from ATCC. All cell lines were cultured in medium as ATCC recommended. All media were supplemented with 10% foetal bovine serum (ExCell Bio, China) and 1% penicillin/streptomycin (NAM Biotech, China). All cell lines were confirmed to be mycoplasma-free, subjected to STR identification for research by Tsingke Biotechnology Co., Ltd. (Beijing, CHINA) using Applied Biosystem® GeneMapper Software 5 over three years.

### Nuclear and cytoplasmic extraction

This study employed the NORGEN kit (Cat. 21000, NORGEN, USA) to isolate nuclear and cytoplasmic fractions. Briefly, 1 × 10^6^ cells were subjected to lysis with Cell Fraction Buffer on ice for a period of 10 min. And then, centrifugation at 5000×*g* for 5 min at 4 °C was performed to collect either the supernatant or the pellet for subsequent purification of cytoplasmic or nuclear fractions, respectively. The RNAs from both fractions were then extracted and subsequently detected using RT–qPCR.

### Fluorescence in situ hybridization (FISH) assay

Probes with 5′-Cy3-AGU AUC UUC CCA AGA GAA AUG ACA GCA GUG UCC ACA CAA A-Biotin modification for MYO16-AS1 were synthesized (RiboBio, China). Following the synthesis of these probes, cells were incubated with them for an overnight duration at 37 °C. The nuclei were then counterstained with DAPI and imaged using a Zeiss LSM 800 laser scanning confocal microscope.

### Actinomycin D assay

A549 and Beas-2B cell lines were subjected to treatment with 10 µg/ml actinomycin D (Selleck Chemical, Houston, USA) for a range of time points spanning 3, 6, 9, and 12 h. After the indicated incubation times, cells were harvested, and the expression levels of MYO16-AS1 were quantitatively analyzed using the RT‒qPCR technique.

### Chromatin immunoprecipitation (ChIP) assay

The ChIP assay was constructed as per the instructions provided in the ChIP Assay kit (Beyotime, China). The cells were initially fixed with 1% formaldehyde for a duration of 10 min and then subsequently slaked with glycine. Following this, the cells were washed with pre-cold PBS and exposed to cell lysis. Subsequently, the crosslinked chromatin was fragmented to a size range of 200–300 bp using sonication. The resultant lysates were incubated with H3K27ac antibodies at a temperature of 4 °C for an overnight period while being gently rocked. The addition of Protein A/G Magnetic beads was then carried out and the complexes were incubated for an additional 60 min. After this, the ChIP complexes were eluted and the DNA concentrate was isolated with the aid of a DNA purification kit. The DNA was finally resuspended in distilled water for downstream analysis. PCR amplification was performed with primer sequences and the resulting PCR products were analyzed via loading onto a 2% agarose gel.

### Plasmid construction, and lentiviral infection

Human MYO16-AS1 transcript was synthesized and cloned into the pLVX-puro vector (Geenseed Biotech, China) and knockdown MYO16-AS1 constructs were cloned in the CRISPR/Cas13 plasmid. All constructs were verified by Sanger sequencing. The negative control was an empty plasmid. Lipofectamine 3000 (Invitrogen, USA) was used to perform the transfection according to the manufacturer's instructions, and total RNA and proteins were collected after 48 h of transfection. The supernatant of cultured 293 T cells was utilized to infect A549 and 1975 cells. The stable cell lines were chosen via 2 µg/ml puromycin (Beyotime, China) selection, and MYO16-AS1 expression was confirmed through reverse transcription-quantitative polymerase chain reaction (RT‒qPCR). All the sequences used in this experiment are presented in Supplemental Table 4.

### Transwell assay

For the migration and invasion assays, a total of 5 × 10^4^ cells were seeded onto the upper layer of a Transwell insert (8 μm, Corning, USA), while the lower chamber contained cell medium supplemented with 10% FBS. To facilitate the invasion assay, a Matrigel matrix (Corning, USA), which was diluted with serum-free RPMI 1640, was added to the upper layer of the transwell membrane at 37 °C for 2 h before cell seeding. The migration assay was incubated at 37 °C for 24 h, while the invasion assay was incubated for 48 h under an atmosphere of 5% CO2. Subsequently, the upper layer of the transwell membrane was wiped, and the Transwell membrane was fixed using 4% paraformaldehyde. To visualize the cells, the Transwell membrane was stained with 0.1% crystal violet and imaged using an Olympus CKX53 inverted microscope.

### Animal studies

Six female BALB/c nude mice (Vital River, China), aged four weeks old, were included per group for constructing subcutaneous tumor formation models. The stable cell lines transfected with the MYO16-AS1 KD virus and the corresponding NC virus were successfully established. A cell suspension of 0.1 ml containing 1 × 10^6^ stable cells (A549-MYO16-AS1 KD, A549-MYO16-AS1 KD NC) was subcutaneously injected into the axilla of the forelimb. The tumor size was monitored every week for 35 days after macroscopic visibility was established. Before injection, the mice were randomized into groups, and the tumor volume was calculated using the formula (width2 × length)/2. All in vivo experiments were conducted in strict compliance with the ethical guidelines of the Committee on the Ethics of Animal Experiments of The Second Hospital of Shandong University.

### RNA extraction and RT‒qPCR

TRIzol (Invitrogen, USA) was utilized to isolate total RNA from LUAD tissues and cells following the manufacturer’s instructions. The obtained cDNAs of lncRNA and mRNA were synthesized by the lnRcute First-Strand cDNA Kit (TIANGEN, Beijing, China). Real-time qPCR analyses of mRNA were conducted using the lnRcute lncRNA qPCR Kit (SYBR Green, TIANGEN, Beijing, China) on a QuantStudio^TM5^ Real-Time PCR System (Thermo Scientific, USA). The 2^−△△CT^ method was used to calculate the differences, and β-Actin served as the endogenous control for normalization.

### Western blot assay

Cells were lysed in RIPA (Beyotime, China) lysis buffer supplemented with proteinase and phosphatase inhibitors. The protein concentrations were determined by a multifunctional enzyme-linked analyzer (BioTek, USA) at 562 nm using a BCA assay (Beyotime, China). Subsequently, protein samples (50 μg) were separated on an SDS-PAGE gel and transferred onto polyvinylidene fluoride (PVDF) membranes (Millipore, Burlington, MA, USA). The membranes were incubated overnight at 4 °C with the appropriate primary antibody, followed by incubation with HRP-linked secondary antibodies for 2 h. The visualization and analysis of the signals were performed using a Tanon 5200 system (Tanon, China). Detailed information regarding the antibodies used in this study is provided in Supplemental Table 5.

### Dual-luciferase reporter assay

To generate dual luciferase reporter plasmids, which harbored the HK2 3′ UTR region encompassing the IGF2BP3 binding sites, we utilized a pMIR-Reporter vector (Tsingke, China). The resulting constructs were then co-transfected with pRL-TK plasmids into A549 cells using Lipofectamine 3000 (Invitrogen, Carlsbad, CA, USA). After 24 h, luciferase activities were determined using a Luciferase Reporter Assay System (Promega, Madison, WI, USA), and the readings were obtained using Biotek Cytation 5.

### RNA pull-down assay

The biotinylated probes for MYO16-AS1 and NC were synthesized by Tsingke (Beijing, China), and the sequences are presented in Supplemental Table 4. A549 cells were lysed according to the manufacturer’s RNA pull-down kit (Geneseed Bio, Guangzhou, China), and the resulting lysates were incubated with biotinylated probes for MYO16-AS1 and NC along with streptavidin magnetic beads overnight at 4 °C. After hybridization, all beads were washed with a washing buffer, and RNA bound to the magnetic beads was extracted with protein. The pulled-down proteins were analyzed by mass spectrometry, Coomassie blue staining, and Western blotting.

### Statistical analysis

Statistical analyses were carried out with GraphPad Prism (version 6.0) software. Student's t-test was used to assess the statistical significance of differences between experimental groups, while one-way analysis of variance (ANOVA) was employed for comparison among more than two groups. The correlation between measurements was evaluated using Pearson's correlation analysis. All data are expressed as the mean ± standard deviation (SD) of three independent experiments. A *p*-value of less than 0.05 was regarded as statistically significant (*denotes *p* < 0.05; **denotes *p* < 0.01; ***denotes *p* < 0.001).

## Results

### The MYO16-AS1 expression level is notably decreased in human LUAD tissues and cell lines.

**Fig. 1:**
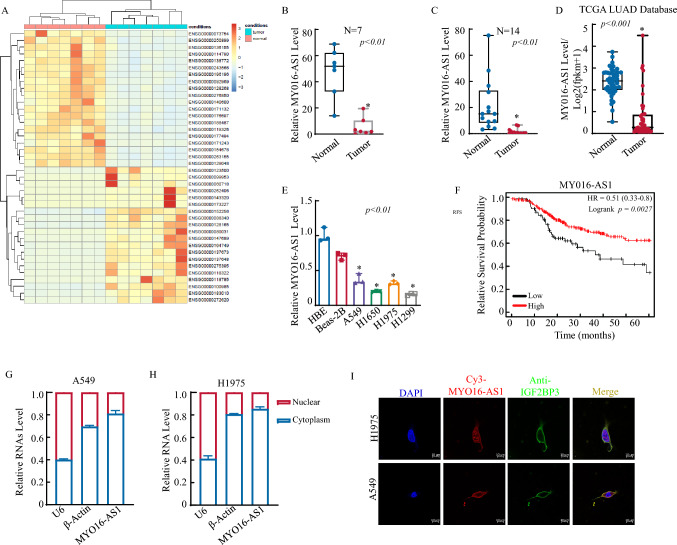
Downregulation of MYO16-AS1 was observed in human LUAD tissues and cell lines. **A** A heatmap of RNAseq (ribo-free) result was generated to show the top DEGs (*n* = 7). **B** A histogram was used to show the level of MYO16-AS1 in the LUAD RNAseq data. The MYO16-AS1 expression levels were analysed by RT‒qPCR from 14 pairs of frozen fresh LUAD and adjacent normal tissue **C** and were analysed from the TCGA database (**D**). **E** Real-time PCR was performed to detect MYO16-AS1 expression, and an asterisk (*) indicates a significant increase from normal HBE cells (*p* < 0.05). **F** The RFS analysis of LUAD patients was performed at kmplot.com. **G** and **H** Cytoplasmic and nuclear RNA extracts were purified from A549 and H1975 cells, and real-time PCR was performed to detect MYO16-AS1 expression. **I** Confocal images were captured using the AxioVision Rel.4.6 computerized image system, and MYO16-AS1 expression levels were analysed with a Cy3-labelled anti-MYO16-AS1 probe. An anti-IGF2BP3 antibody was used to detect the localization of MYO16-AS1 in LUAD cells

It is well-known that lncRNAs play a crucial role in cancer progression[10]. However, the role and mechanism of lncRNAs in LUAD remain unclear. Therefore, 7 pairs of LUAD fresh tissue and adjacent normal tissue were collected for RNA-seq (ribo-free), and bioinformatics analysis is shown in Fig. 1A. Heatmap results showed the top 20 upregulated and 20 downregulated genes with |log2FC|> 1 and *p*-value < 0.05 (Table S 1) in LUAD tissues compared with adjacent normal tissue. The lncRNA MYO16-AS1 gene was one of the most significant differentially expressed genes (DEGs) in LUAD tissue (Fig. 1B). MYO16-AS1, located on the chromosome 13, was expressed mainly in lung tissue (Figure S1A). Furthermore, RT‒qPCR was applied to validate the expression of MYO16-AS1 in LUAD tissue of 14 LUAD patients (including 14 cancer tissues and 14 adjacent normal tissues). As shown in Fig. 1C, MYO16-AS1 expression was significantly downregulated in cancer tissue compared with normal tissue, consistent with the results of noncoding RNA sequencing (Fig. 1B), TCGA database analysis (Fig. 1D) and analysis of the GSE168466 dataset (Figure S1B); furthermore, MYO16-AS1 expression was downregulated at both early and late tumor stages relative to that in normal tissue (Figure S1C). We also measured the MYO16-AS1 expression level in LUAD cell lines (A549, H1299, H1975) and normal epithelial cell lines (16-HBE and Beas-2B). As shown in Fig. 1E, a low expression level of MYO16-AS1 was observed in LUAD cell lines. Moreover, based on the median expression level of MYO16-AS1, we divided the patients into two groups: high expression and low expression; Kaplan‒Meier analysis of the correlation between MYO16-AS1 expression and patient recurrence-free survival (RFS) showed that low expression of MYO16-AS1 was associated with a poorer prognosis than high expression (Fig. 1F). Overall, MYO16-AS1 downregulation was observed in both tissue and cell lines, illustrating that MYO16-AS1 may play an anticancer role in LUAD. In addition, we also interestingly found by cytonuclear extract assay that MYO16-AS1 was mainly expressed in the cytoplasm of A549 and H1975 cells (Fig. 1G, H). Consistent with MYO16-AS1 expression, Fluorescence in situ hybridization (FISH) assay showed that MYO16-AS1 was localized mainly in the cytoplasm (Fig. 1I). Taken together, our results strongly demonstrate that the expression level of MYO16-AS1 is decreased in human LUAD cells in vitro and in human LUAD tissues in vivo.

### MYO16-AS1 overexpression inhibits LUAD cell progression.

**Fig. 2:**
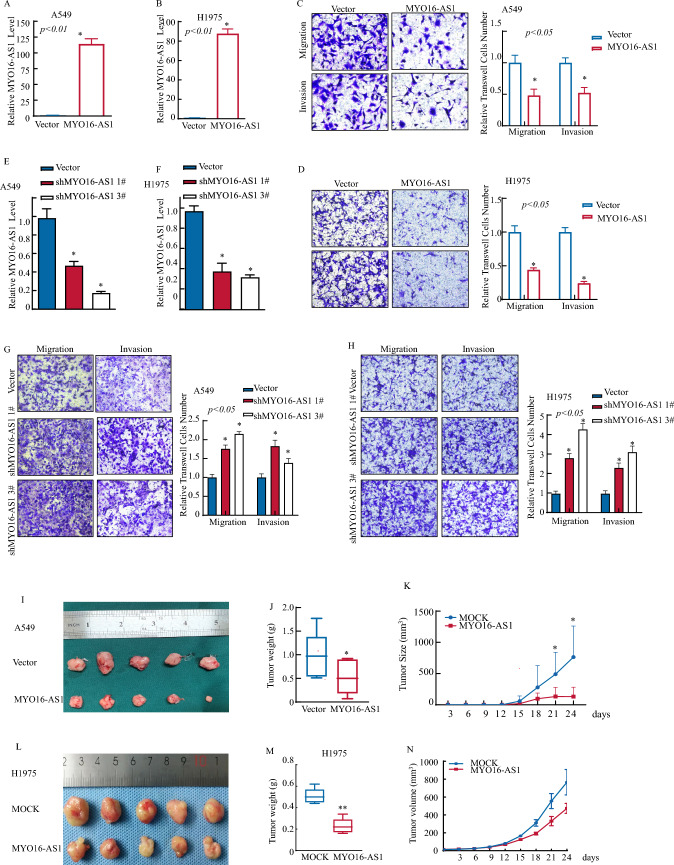
MYO16-AS1 inhibited LUAD progression in vitro and in vivo. **A** and **B** MYO16-AS1 expression was detected by RT-qPCR in A549 and H1975 cells after transfection with MYO16-AS1 overexpression lentivirus, and an asterisk (*) indicates a significant increase from the vector control (*p < 0.05). **C** and **D** Transwell assays of A549 and H1975 cells overexpressing MYO16-AS1 were performed. The migrated and invaded cells were photographed under an Olympus DP71 microscope, and the number of cells in each image was counted with ImageJ software. **E** and **F** MYO16-AS1 expression was detected by RT‒qPCR in A549 and H1975 cells after transfection with shMYO16-AS1 lentivirus, and an asterisk (*) indicates a significant increase from the vector control (*p < 0.05). **G** and **H** Transwell assays of A549 and H1975 cells were performed in MYO16-AS1-knockdown cells. The migrated and invaded cells were photographed under an Olympus DP7. The invasion rate was normalized to the insert control according to the manufacturer’s instructions. **I**–**N** Cell-derived xenografts overexpressing MYO16-AS1 and vector control A549 and H1975 cells were injected into nude mice, and the tumor weight and tumor size were measured to evaluate the degree of malignancy after overexpression of MYO16-AS1, *p < 0.05

To determine the underlying function of MYO16-AS1 in LUAD development, the MYO16-AS1 overexpression viral vector and the corresponding control vector were transduced into A549 and H1975 cells separately (Fig. 2A, H). MYO16-AS1 overexpression notably reduced the migration and invasion activity of LUAD A549 (Fig. 2C) and H1975 (Fig. 2D) cell lines in transwell assays. In addition, we also performed shRNA-targeted MYO16-AS1 in A549 and H1975 cells (Fig. 2E, F). Compared with those in the vector control cells, the migration and invasion abilities were increased in shRNA MYO16-AS1 knockdown cells, as shown in Fig. 2G, H. A nude mouse xenograft tumor model was established to determine the effect of MYO16-AS1 in vivo (Fig. 2I-N), and the results showed that stable overexpression of MYO16-AS1 significantly decreased the size, volume, and weight of subcutaneous tumors in nude mice compared that of those in the control group (Fig. 2l-N). Taken together, these results indicated that MYO16-AS1 plays a tumor suppressor role in LUAD invasion and migration in vitro and in vivo.

### MYO16-AS1 interacts with the IGF2BP3 protein located in the cytoplasm in LUAD cells.

**Fig. 3:**
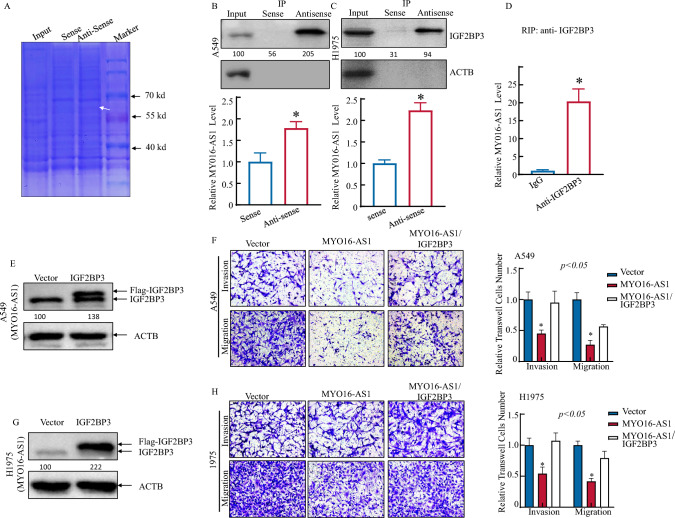
The interaction of MYO16-as1 with the IGF2BP3 protein directly inhibited the migration and invasion of A549 and H1975 cells. **A** Coomassie blue staining of SDS‒PAGE gel was performed to show the binding protein using biotin-labelled sense and anti-sense probes of MYO16-AS1. **B** and **C** RNA pull-down assay by biotin-labelled sense and antisense MYO16-AS1 probes was performed in A549 and H1975 cells, and then the IGF2BP3 protein/RNA complex was used to test IGF2BP3 levels, and the positive control was tested by RT‒qPCR. **D** RIP assay by IGF2BP3 antibodies was performed in A549 cells, and then the IGF2BP3 protein/RNA complex was used to test MYO16-AS1 levels. The asterisk (*) indicates a significant increase from the sense control. **E-H** Flag-tagged IGF2BP3 lentivirus was stably transfected into A549 (MYO16-AS1) and H1975 (MYO16-AS1) cells. Western blotting was used to detect the overexpression efficiency of the IGF2BP3 protein. Transwell assays of A549 (MYO16-AS1/IGF2BP3) and H1975 (MYO16-AS1/IGF2BP3) cells were performed. The migrated and invaded cells were photographed under an Olympus CKX53 inverted microscope, and the number of cells was counted with ImageJ software

Accumulating evidence indicates that cytoplasmic lncRNAs participate in different levels of gene regulation by interacting with RNA-binding proteins (RBPs) to disturb mRNA and protein stability, translation efficiency, sponging of cytosolic factors and miRNAs, and alteration of cell recognition and signaling pathways [11, 12]. To assess the possible interaction between MYO16-AS1 and RBPs in LUAD cells, a biotin-labeled antisense MYO16-AS1 probe was used to pull-down the interacting proteins in A549 cells, and the silver staining results are shown in Fig. 3A. These results combined with the mass spectrometry results (Table S2) indicate that MYO16-AS1 may interact with the IGF2BP3 protein. Subsequently, we unexpectedly found that MYO16-AS1 could bind with IGF2BP3 via an RNA pull-down assay in A549 and H1975 cells (Fig. 3B, C). Accordingly, the IGF2BP3 protein could bind to MYO16-AS1, as determined through an RNA immunoprecipitation (RIP) experiment (Fig. 3D). The confocal images shown in Fig. 1I also confirmed that MYO16-AS1 and IGF2BP3 are co-localized in the cytoplasm of cancer cells. These results provided robust evidence of the direct binding of MYO16-AS1 to IGF2BP3. However, whether the downstream function of the MYO16-AS1/IGF2BP3 complex is regulated is still unknown. To address this knowledge gap, the IGF2BP3 overexpression construct was transfected into A549 and H1975 MYO16-AS1 cells (Fig. 3E, G, Fig. S2A, B). Transwell experiments showed that IGF2BP3 overexpression reversed the inhibition of LUAD cell migration and invasion mediated by MYO16-AS1 (Fig. 3F, H). Our results above demonstrate that MYO16-AS1 inhibits malignant activity in LUAD cells and that these effects can be reversed by IGF2BP3 overexpression.

### MYO16-AS1-mediated glucose metabolism reprogramming via HK2 is essential for human NSCLC cell invasion.

**Fig. 4:**
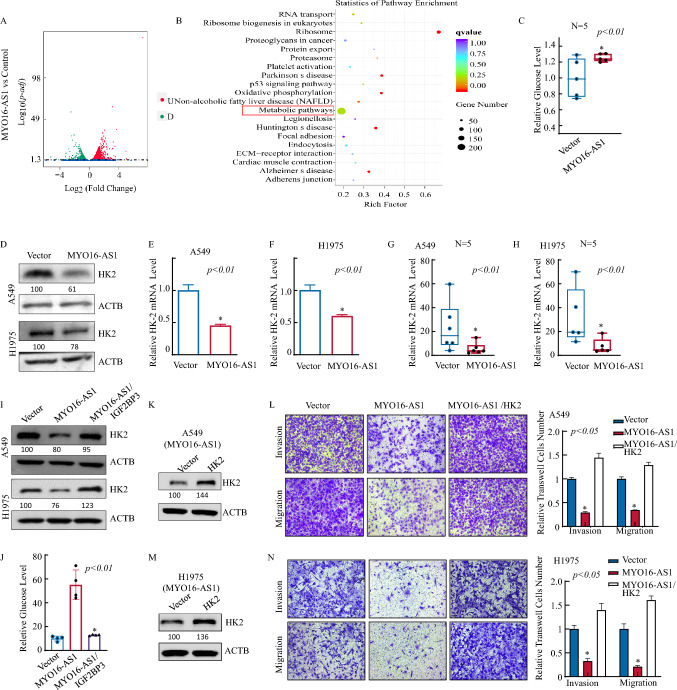
MYO16-AS1-mediated glucose metabolism reprogramming via HK2 was essential for the invasion of human NSCLC cells. **A** Volcano plot showing the DEGs due to MYO16-AS1 overexpression in LUAD cells. **B** KEGG enrichment analysis for KEGG pathways in MYO16-AS1-inhibited genes. **C** Relative glucose levels in xenograft tumor s derived from A549 MYO16-AS1cells. **D** Western blotting assay and **E–F** RT-qPCR assay were performed to detect the expression of HK2 in A549 and H1975 cells with MYO16-AS1 overexpression and vehicle control. **G** and **H** Relative HK2 levels in xenograft tumor s derived from A549 MYO16-AS1 and H1975 MYO16-AS1 cells. **I** and **J** Western blot and RT-qPCR analyses were performed to evaluate the expression of HK2 in A549 and H1975 cells with MYO16-AS1 overexpression, MYO16-AS1-overexpressing cells with overexpression of IGF2BP3 and vehicle control-treated cells. **K-N** Overexpression constructs of HK2 were stably transfected into A549 and H1975 MYO16-AS1-overexpressing cells. Western blotting was used to detect the knockdown efficiency of HK2 protein levels. The invasion and migration abilities of the indicated cells were evaluated. Student’s *t-*test was utilized to determine the *p-*value; an asterisk (*) indicates a significant increase in comparison to A549 and H1975 (Vector) transfectants (*p < 0.05)

The above findings demonstrate the inhibitory effect of MYO16-AS1 on LUAD both in vitro and in vivo. However, the precise molecular mechanism underlying this effect remains to be elucidated. To further understand the regulatory network of MYO16-AS1 in LUAD cells, we performed RNA-seq analysis, which revealed that MYO16-AS1 overexpression affects several signaling pathways, including the P53 pathway, focal adhesion pathway, NF-κB signaling, as well as the metabolic pathway (Fig. 4A, B, Figure S3A, Table S3). These pathways have been previously reported to play a vital role in cancer progression and development. Furthermore, we observed that the glucose level was significantly higher in MYO16-AS1-overexpressing cells in the A549 mouse model (Fig. 4C), which suggests that MYO16-AS1 may play a role in impairing glucose metabolism in LUAD cells. The identified oncogenic pathways and altered metabolic function warrant further investigation to unravel the precise molecular mechanisms underlying MYO16-AS1’s impact on LUAD. To explore the key genes in the regulation of the metabolic pathway, together with the direct binding relationship of MYO16-AS1 and IGF2BP3 in Fig. 3, intersection analysis between the IGF2BP3 iCLIP results and downregulated genes of RNA-seq data showed that HK2 was one of the downregulated genes in MYO16-AS1-expressing lung cells (Figure S3C). Not surprisingly, we observed decreases in HK2 protein and RNA levels (Fig. 4D–F), with consistent results in mouse models in vivo (Fig. 4G, H). In addition, IGF2BP3 overexpression in MYO16-AS1 cells restored HK2 expression in A549 and H1975 cells (Fig. 4I), and glucose level also reversed after IGF2BP3 overexpressed in MYO16-As1 overexpression cells (Fig. 4J). These results illustrated that the MYO16-AS1/IGF2BP3 complex may be involved in HK2 expression regulation in LUAD cells, in turn contributing to glucose metabolism disorganization. We further transduced the lentiviral HK2 overexpression plasmid into MYO16-AS1-overexpressing cells (Fig. 4K, M), and subsequent invasion and migration assays (Fig. 4L, N) showed that exogenous expression of HK2 restored the inhibitory effects of MYO16-AS1 in LUAD cells. These results reveal that MYO16-AS1-mediated HK2 downregulation is the key event underlying MYO16-AS1-mediated inhibition of LUAD cell invasion.

### MYO16-AS1 competitively bind to IGF2BP3 with HK2 RNA, contributing to HK2 mRNA degradation.

**Fig. 5:**
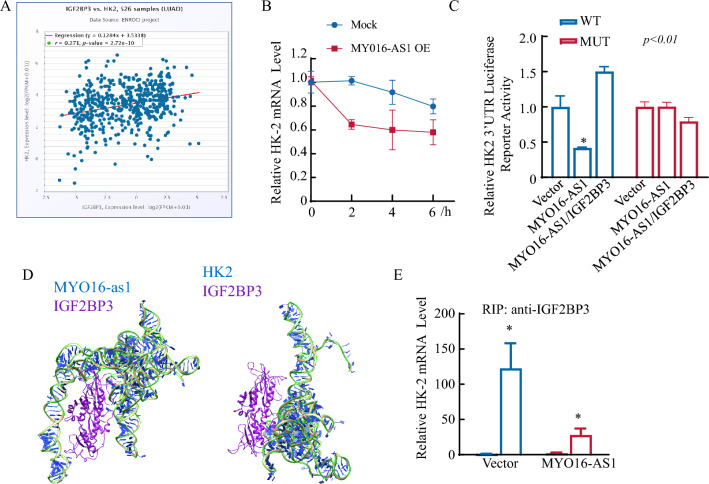
MYO16-AS1 competes with IGF2BP3 for HK2 RNA-binding, contributing to HK2 mRNA degradation. **A** The correlation of IGF2BP3 and HK2 mRNA levels in the TCGA database. **B** The HK2 mRNA stability assay by RT‒qPCR was performed in A549 (MYO16-AS1 and vector) cells. **C** An HK2 3’UTR luciferase reporter assay was performed to detect the regulation of MYO16-AS1 and IGF2BP3 transfection. **D** Graphical illustration showing the predicted interaction between the MYO16-AS1 RNA and the IGF2BP3 protein in the HDOCK server. **E** HEK293T cells were co-transfected with the indicated expression plasmids for 36 h. Cells were subjected to RIP/RT‒qPCR analyses

The above findings revealed that MYO16-AS1 overexpression impairs human LUAD progression as a result of MYO16-AS1 competing with HK2 mRNA for binding to IGF2BP3. In exploring the mechanism by which MYO16-AS1 regulates HK2, we first found the relationship between IGF2BP3 and HK2 mRNA in Fig. 5A by utilizing the starBase database, and the results showed that these two proteins are weakly correlated (*R* = 0.271). The RNA stability results also revealed that HK2 mRNA was destabilized due to MYO16-AS1 overexpression (Fig. 5B). The HK2 mRNA 3′-UTR-driven luciferase reporter also consistently elevated in MYO16-AS1-overexpressing LUAD cells and was significantly lower than that in vehicle control cells (Fig. 5C), and exogenous transfection of IGF2BP3 in MYO16-AS1-overexpressing cells abolished the HK2 mRNA 3′UTR inhibition ability (Fig. 5C). Furthermore, the IGF2BP3 binding site mutation lost the inhibition of MYO16-AS1 overexpression. Furthermore, the HDOCK server was used to predict the relationship between MYO16-AS1/IGF2BP3 and IGF2BP3/HK2 mRNA (Fig. 5D) and indicated potential competition between MYO16-AS1 and HK2 mRNA. A RIP assay was subsequently performed to assess whether MYO16-AS1 can compete with HK2 for binding to IGF2BP3 (Fig. 5E). IGF2BP3 was found to directly bind to HK2, and this binding was inhibited by ectopic expression of MYO16-AS1 (Fig. 5E). Taken together, MYO16-AS1 competitively bind to IGF2BP3 with HK2 RNA, contributing to HK2 mRNA degradation.

### MYO16-AS1 downregulation was attributed to histone modification of its promoter region.

**Fig. 6:**
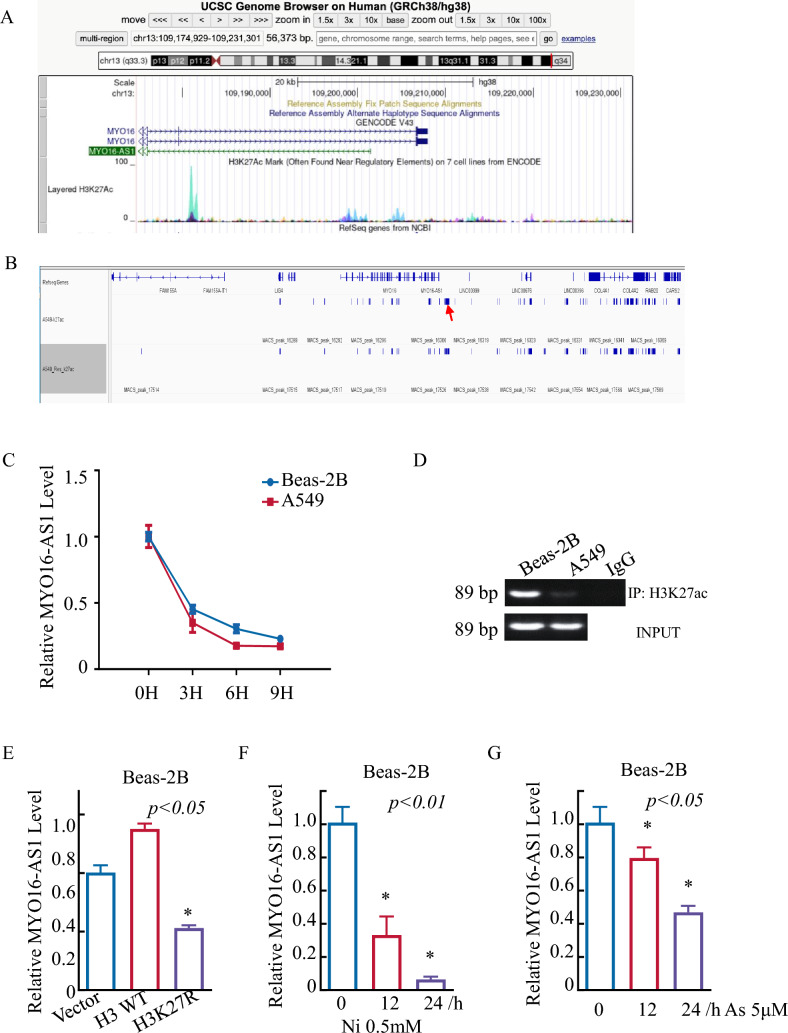
MYO16-AS1 downregulation was attributed to histone modification of its promoter region. **A** This peak is located on chromosome 13 and annotated to the MYO16-AS1 promoter region. The figure also shows the location of all H3K27ac peaks in this region, hyper and hypoacetylated peaks (FDR < 0.05), as well as the -log10(P) value and log (fold change) values for the normalized read count differences for each peak, calculated using a quasi-likelihood F test. Data were obtained from the UCSC Genome Browser. 'FDR neg' and 'FDR pos' indicate hypo- and hyperacetylated peaks, respectively, FDR < 0.05. **B** The GEO database showed the peaks of the MYO16-AS1 promoter region. **C** The MYO16-AS1 mRNA stability assay by RT‒qPCR was performed in Beas-2B and A549 cells. **D** ChIP-PCR analysis of H3K27ac binding to MYO16-AS1 gene promoter regions. **E** RT‒qPCR analysis of MYO16-AS1 transfection with the indicated expression plasmids. **F** and **G** RT‒qPCR assays were performed to detect MYO16-AS1 expression in Beas-2B cells treated with nickel and arsenic. **I** The proposed mechanisms underlying MYO16-AS1 inhibition of human LUAD cell proliferation, migration and invasion in vitro and in vivo

Chromatin accessibility is often associated with tumor development, and histone modifications are one of the important reasons for accessibility alterations[13]. Consistent with this observation, the UCSC Genome Browser database shows that the regulation of MYO16-AS1 is a highly possible result of H3K27ac modification (Fig. 6A). Moreover, H3K27ac CHIP data from the GEO database (GSE96780) revealed that MYO16-AS1 may be regulated at the transcriptional level in lung cancer tissues (Fig. 6B). To further verify the mechanism regulating MYO16-AS1 expression, we first excluded the possibility of RNA stability-mediated regulation of MYO16-AS from the result shown in Fig. 6C, in which both Beas-2B and A549 cells were treated with Act-D. The CHIP-PCR was used to understand H3K27ac-mediated MYO16-AS1 downregulation in LUAD, and the results showed that H3K27ac levels were markedly reduced in A549 cancer cells compared with Beas-2B normal cells (Fig. 6D), meanwhile, H3.1 WT and H3K27R constructs were transfected into A549 cells; MYO16-AS1 was upregulated with ectopic expression of H3.1 WT and downregulated in H3K27R cells compared with WT A549 cells (Fig. 6E). Heavy metals (Nickel, Arsenic. etc.) contribute to the malignant transformation of lung epithelial cells due to dysregulation of histone modification [14]. Moreover, MYO16-AS1 RNA expression was obviously reduced after nickel and arsenic treatment (Fig. 6F, G). These results strongly suggest that an epigenetic mechanism mediates MYO16-AS1 downregulation in LUAD cells.

## Discussion

LncRNAs undoubtedly play a central role in tumor invasion, stemness maintenance, and chemoresistance[15, 16]. However, the molecular mechanism by which lncRNAs control LUAD progression via metabolic pathways is still largely unclear. Herein, we found that MYO16-AS1, a novel lncRNA expressed mainly in lung tissue, was downregulated in LUAD tissues and cell lines and that low expression of MYO16 is strongly associated with the severity and poor prognosis of lung cancer [17]. This is consistent with previous bioinformatics studies that reported lncRNAs, including MYO16-AS, as potential prognostic biomarkers in LUAD [18]. Moreover, the ectopic expression of MYO16-AS1 functionally inhibited LUAD progression in vitro and in vivo*,* revealing its tumor suppressor function in LUAD. The mechanistic study revealed that histone modification in the MYO16-AS1 promoter region leads to the downregulation of MYO16-AS1 and competition with IGF2BP3, in turn impairing HK2 mRNA stability and ultimately resulting in LUAD malignant progression. Taken together, our research provides novel insight into the underlying mechanism of MYO16-AS1-mediated suppression of LUAD progression.

MYO16-AS1 was discovered to be expressed mainly in lung tissue and to be downregulated in LUAD; moreover, recent studies have reported the expression level of MYO16-AS1 in Parkinson's disease [19] and bladder cancer, but the regulatory mechanism has not been explored. Here, we revealed the antitumor role of MYO16-AS1 in LUAD and its potential as a biomarker for LUAD diagnosis. This regulatory relationship between MYO16-AS1 and IGF2BP3 might be present mainly in tumor epithelial cells rather than mesenchymal cells or immune cells. In addition, the subcellular localization of lncRNAs mainly determines their biological function. For example, LincRNA-p21\GAS5\MALAT1, which are located in the cytoplasm and act as competing endogenous (ceRNAs) or interact with RBPs, could enhance mRNA stabilization and translation in the context of cancer progression [20–22]. In this project, MYO16-AS1 was found to inhibit HK2 mRNA degradation through competitive binding with IGF2BP3 protein in the cytoplasm, inhibiting tumor progression both in vitro and in vivo. It is well-known that dysregulation of glucose metabolism in cancer nests contributes to the absence of oxygen and glycolysis[23]. HKs, as the first rate-limiting step of glycolysis [24], are a possible target for cancer therapy. Here, we identified that HK2 was overexpressed in LUAD, which was consistent with previous studies [25]. Some studies have reported that HK2 has great advantages as a therapeutic target for cancer; however, HK2 has limitations as a therapeutic target due to its widespread expression in all organs[26]. Thus, MYO16-AS1 may be a better potential therapeutic target.

Epigenetic modification is a well-known type of gene transcriptional regulation, including lncRNA expression[27]. The UCSC database displayed histone modification of the MYO16-AS1 promoter region, which was very close to the transcription start site of MYO16-AS1. Our research findings determined the regulatory mode of MYO16-AS1 transcription. On the other hand, heavy metals (nickel, arsenic, etc.) and air pollution are key factors in the malignant transformation of lung cells and can inhibit histone H3K27 activity. Herein, we also found that MYO16-AS1 downregulation could attribute to nickel and arsenic.

## Conclusion

In summary, we provide new insight into the relationship between the lncRNA MYO16-AS1 and tumor metabolism and shed light on its regulatory mechanisms in LUAD. Moreover, MYO16-AS1 decreases HK2 mRNA stability through competitive binding with IGF2BP3, enriching the regulation of lncRNA genes and RBPs. These results hold significant implications for the development of a specific therapeutic strategy for LUAD patients targeting MYO16-AS1 and HK2, a key player in tumor metabolism.

## Supplementary Information

Below is the link to the electronic supplementary material.

Supplementary file1 (XLSX 4869 kb)

Supplementary file2 (PDF 602 kb)

## Data Availability

Enquiries about data availability should be directed to the authors.
