# Supplementary figures and images for: Disrupting of IGF2BP3-stabilized HK2 mRNA by MYO16-AS1 competitively binding impairs LUAD migration and invasion
Source: Mol Cell Biochem. 2023 Dec 2;479(10):2795–808. doi: 10.1007/s11010-023-04887-w (PMC11455711; doi:10.1007/s11010-023-04887-w)

Figure S1

A

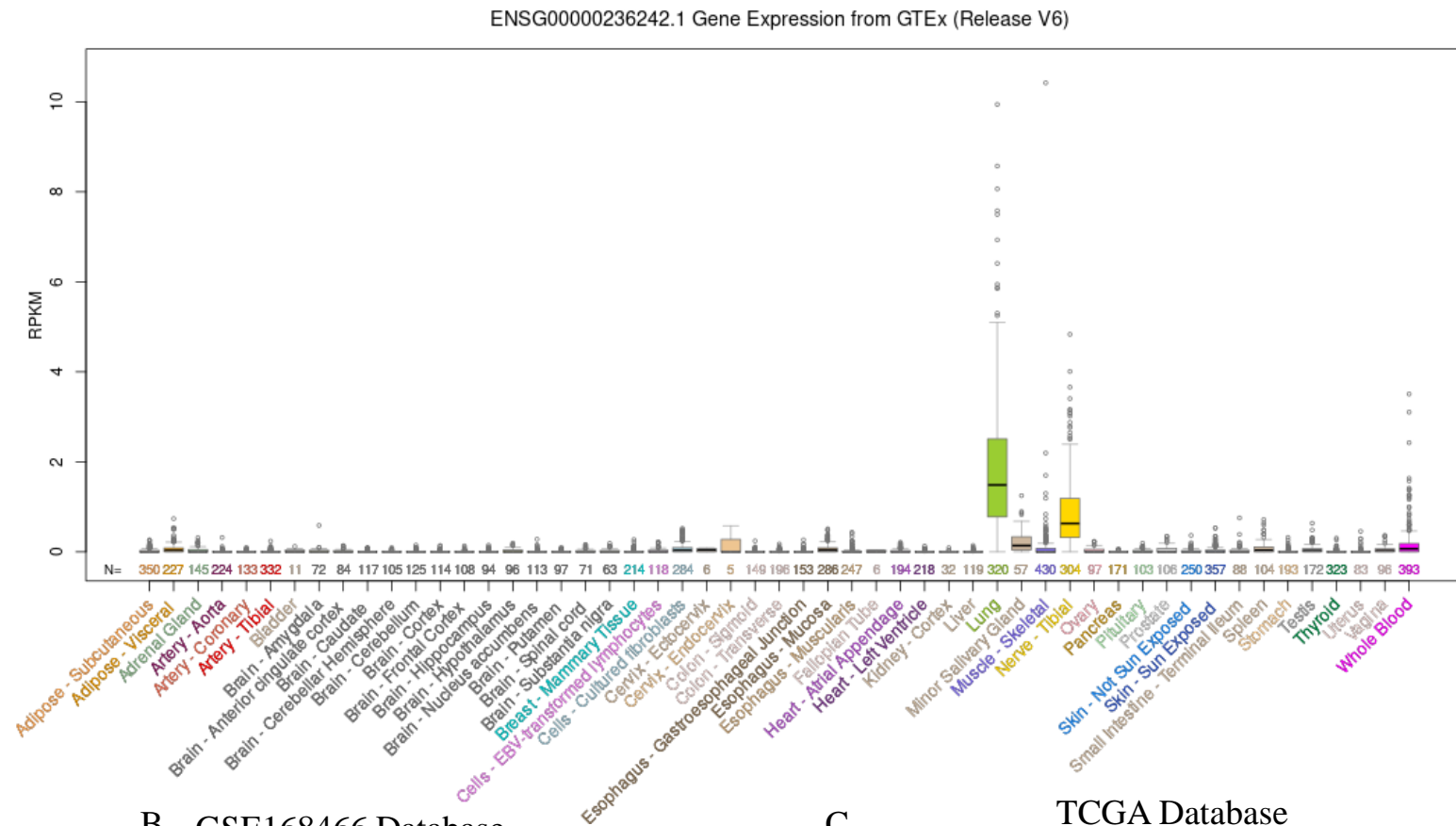

B GSE168466 Database

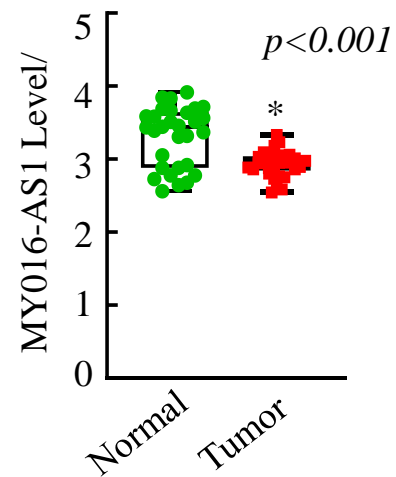

C

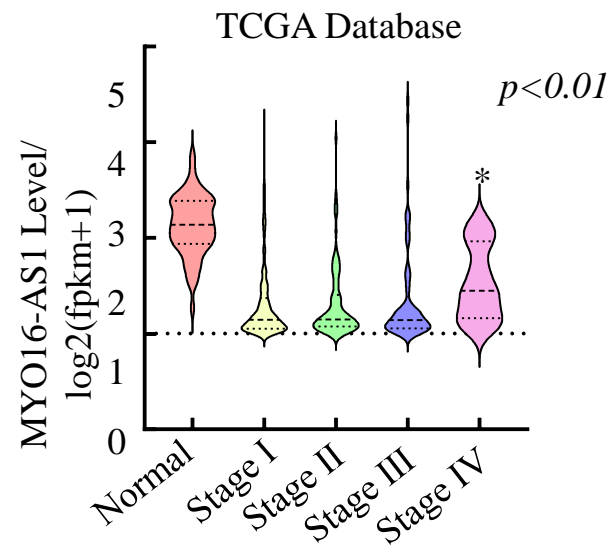

Figure S2

A

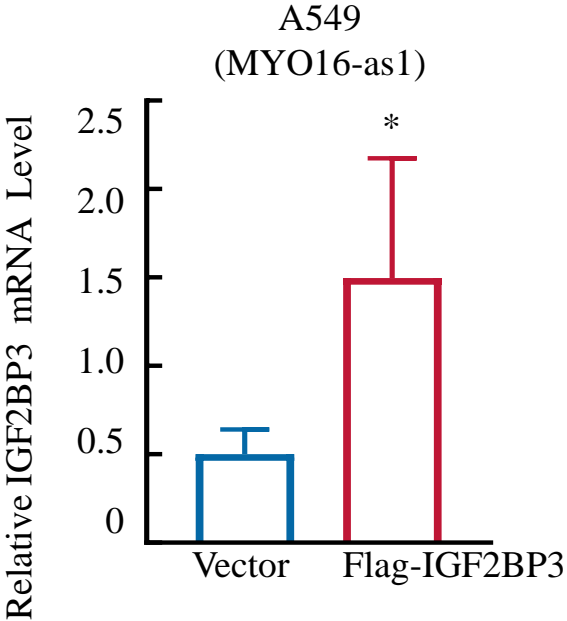

B

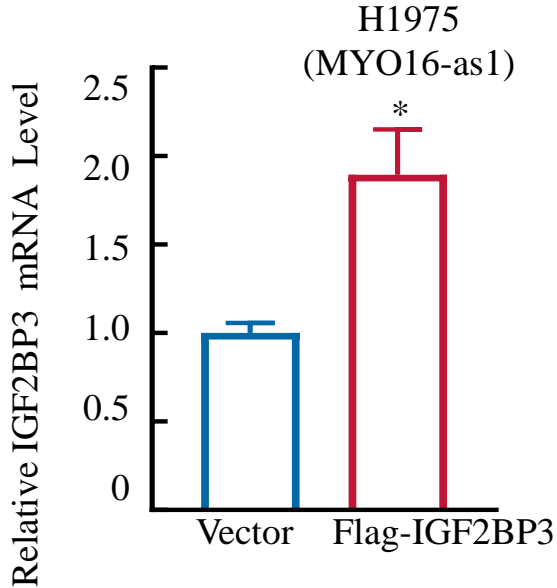

Figure S3

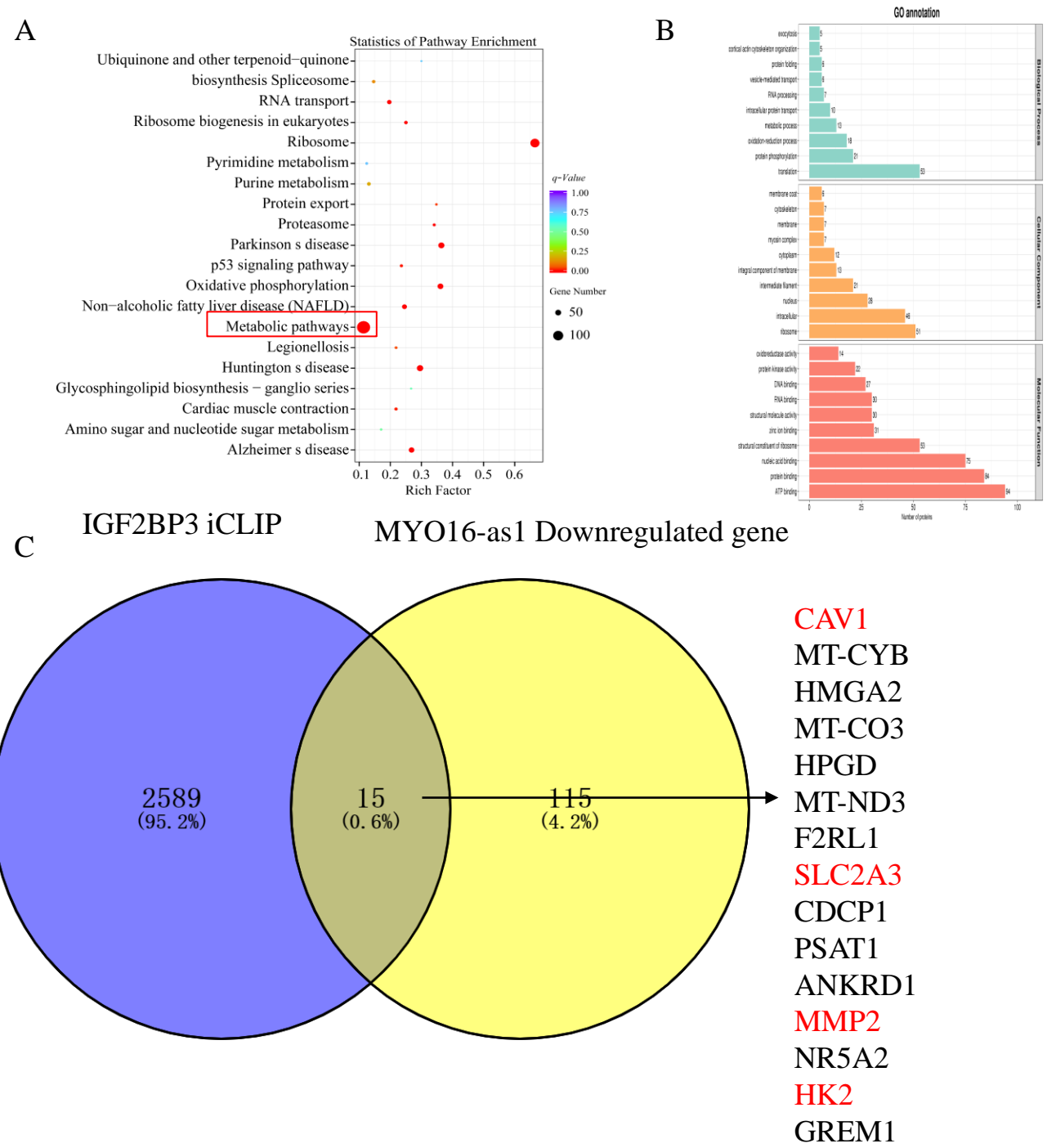

Supplement: Supplementary file 2 — Supplementary file2 (PDF 602 kb) [file 11010_2023_4887_MOESM2_ESM.pdf]
